# Polysomnogram outcomes in patients with laryngomalacia and obstructive sleep apnoea treated surgically versus non-surgically

**DOI:** 10.1017/S0022215123000932

**Published:** 2024-04

**Authors:** Nicolas J Casellas, Shalini Shah, Saiganesh Ravikumar, Nathan D Vandjelovic, John Faria, Paul D Allen, Margo K McKenna Benoit

**Affiliations:** 1 Department of Otolaryngology, University of Rochester Medical Center, Rochester, NY, USA; 2 University of Rochester School of Medicine and Dentistry, Rochester, NY, USA

**Keywords:** Laryngomalacia, polysomnography, obstructive sleep apnea, pediatrics

## Abstract

**Objective:**

To compare supraglottoplasty versus non-surgical treatment in children with laryngomalacia and mild, moderate and severe obstructive sleep apnoea.

**Methods:**

Patients were classified based on their obstructive apnoea hypopnoea index on initial polysomnogram, which was compared to their post-treatment polysomnogram.

**Results:**

Eighteen patients underwent supraglottoplasty, and 12 patients had non-surgical treatment. The average obstructive apnoea hypopnoea index after supraglottoplasty fell by 12.68 events per hour (*p* = 0.0039) in the supraglottoplasty group and 3.3 events per hour (*p* = 0.3) in the non-surgical treatment group. Comparison of the change in obstructive apnoea hypopnoea index in the surgical versus non-surgical groups did not meet statistical significance (*p* = 0.09).

**Conclusion:**

All patients with laryngomalacia and obstructive sleep apnoea had a statistically significant improvement in obstructive apnoea hypopnoea index after supraglottoplasty irrespective of obstructive sleep apnoea severity, whereas patients who received non-surgical treatment had more variable and unpredictable results. Direct comparison of the change between the two groups did not find supraglottoplasty to be superior to non-surgical treatment. Larger prospective studies are recommended.

## Introduction

Laryngomalacia is both the most common congenital laryngeal anomaly and the most common cause of stridor in neonates and infants.^1,2^ Laryngomalacia is typically diagnosed with flexible laryngoscopy, as a collapse of the structures in the laryngeal inlet with poor structural support and tone. Severe cases may lead to failure to thrive and increasing respiratory distress. However, currently there is no objective method for classifying severity of laryngomalacia, and the patient's signs and symptoms are usually used to describe the severity of laryngomalacia and determine appropriate therapy.^3,4^ Holinger *et al*. first reported supraglottoplasty as an effective treatment for patients with severe laryngomalacia.^5^ However, surgical outcomes are variable, and indications for surgery can vary considerably between surgeons.^3,6–8^ For instance, Faria *et al*. found no significant difference when comparing the growth of infants with moderate to severe laryngomalacia who underwent supraglottoplasty to the growth of those treated with medical therapy alone.^3^ Several studies have proposed classification types for laryngomalacia, however the type of laryngomalacia does not seem to predict the outcome of supraglottoplasty.^9^

The natural course of obstructive sleep apnoea in children with laryngomalacia is still unknown. In most cases, symptoms gradually resolve by 6–18 months of age. However, it has been noted that obstructive symptoms may persist even after stridor resolves.^3,10^ The prevalence of obstructive sleep apnoea in laryngomalacia has been reported to be 3–77.4 per cent.^11–14^

Treatment modalities and factors affecting treatment outcomes in children with dual conditions are not thoroughly understood. Several retrospective studies and meta-analyses in children have shown the efficacy of supraglottoplasty in children with congenital laryngomalacia and obstructive sleep apnoea.^8,13,15–17^ Very few, if any, of these studies included an observational control group or evaluated treatment outcomes by obstructive sleep apnoea severity. Previous studies focused on patients with severe obstructive sleep apnoea and laryngomalacia.^8,13,18^ The benefit of supraglottoplasty in patients with mild or moderate obstructive sleep apnoea and clinical evidence of laryngomalacia remains inconclusive.^1,19,20^

The aim of this study was to compare the outcomes of supraglottoplasty versus non-surgical treatment in neonates and infants with diagnosis of laryngomalacia and mild, moderate or severe obstructive sleep apnoea.

## Materials and methods

### Study design

A retrospective chart review was conducted in patients evaluated at Golisano Children's Hospital at the University of Rochester Medical Center (URMC) between 1 January 2014 and 31 December 2019. The TriNetX (https://trinetx.com) search (January 2020) included all patients up to 3 years of age, with a diagnosis of congenital laryngomalacia and a concomitant diagnosis of obstructive sleep apnoea confirmed by polysomnogram. A flexible or rigid laryngoscopy confirmed the diagnosis of laryngomalacia. Patient charts were individually assessed for medical and surgical history. Subjects were classified based on intervention history: supraglottoplasty versus no surgery. Patients were also stratified by severity of obstructive sleep apnoea on the original polysomnogram (mild, moderate, severe). The study was approved by the Institutional Review Board at the University of Rochester Medical Center.

**Figure 1.**
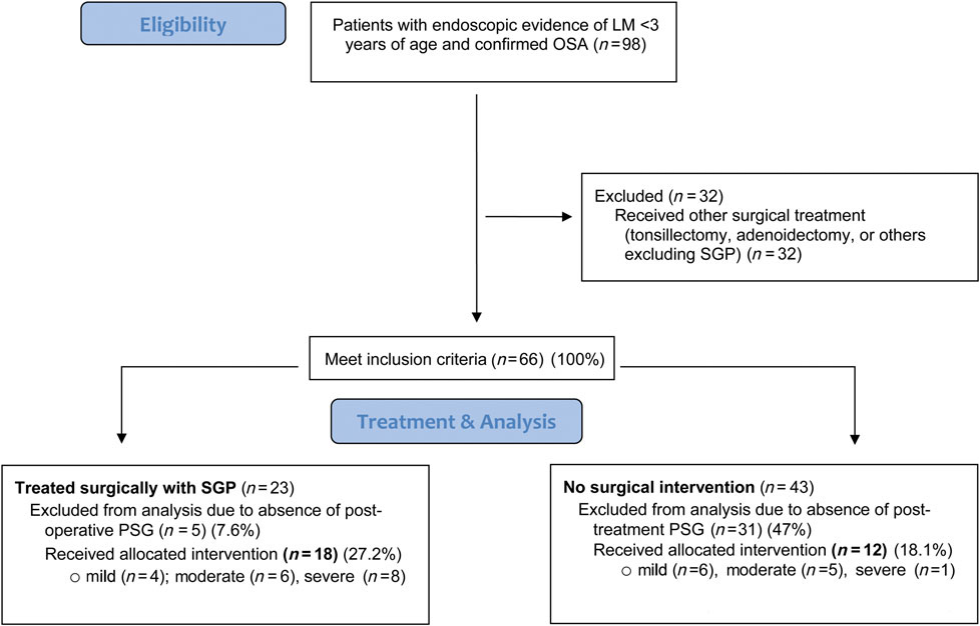
Study population. LM = laryngomalacia; OSA = obstructive sleep apnoea; SGP = supraglottoplasty; PSG = polysomnography

The inclusion criteria for the surgical group required (1) presence of a pre-operative and post-operative polysomnogram, and (2) presence of an operative note indicating the performance of supraglottoplasty. Inclusion criteria for the non-surgical treatment required (1) presence of two polysomnograms, and (2) documentation of observation alone or medical therapy for laryngomalacia and obstructive sleep apnoea. All patients without either a pre- or post-operative polysomnogram were excluded from the study (Figure 1).

Patients in both treatments were seen by the same otolaryngologists during follow-up visits. Patients in the non-surgical treatment group received either observation alone or medical treatment with nasal steroids once daily in each nostril, montelukast daily and/or anti-reflux medications (histamine type 2 antagonist/blocker twice daily or proton pump inhibitor daily), prescribed either by the otolaryngology or sleep medicine providers, or by the patient's primary care provider. Supraglottoplasty was performed by two attending paediatric otolaryngologists, and the decision to proceed with supraglottoplasty was based on the surgeon's overall clinical judgement, results from polysomnography and shared decision making with parents and families. Some subjects who were potential candidates for supraglottoplasty based on severity of laryngomalacia or polysomnogram findings were treated conservatively based on shared decision making between the parents and the surgeon. Therefore, both groups were relatively homogeneous with similar obstructive sleep apnoea severities and co-morbidities.

Information on demographics, medical co-morbidities, secondary airway lesions, other surgical interventions and medical procedures was collected from the electronic medical record. To analyse the data in a standard manner, we used weight percentiles based on growth charts recommended by the Centers for Disease Control and Prevention, according to the respective age and gender of the patient.^21^

### Polysomnography

Comprehensive polysomnograms were performed at a certified paediatric sleep laboratory. Polysomnogram data were scored and interpreted based on rules, terminology and technical specification for the scoring of sleep and associated events, as published in the American Academy of Sleep Medicine Manual.^22^ Collected data included the obstructive apnoea-hypopnoea index (obstructive apnoea-hypopnoea index events per hour), O_2_ nadir (per cent) and central apnoea (events per hour), as well as weight and age on the day of each sleep study to calculate the weight percentile change. The apnoea-hypopnoea index was defined as the total number of obstructive apnoeas, mixed apnoeas and hypopnoeas per hour of total sleep time. Obstructive sleep apnoea severity was defined based on obstructive apnoea-hypopnoea index severity: mild (1–5 events per hour), moderate (> 5–10 events per hour) and severe (> 10 events per hour). Surgical success was defined according to published literature on paediatric obstructive sleep apnoea surgery as apnoea-hypopnoea index decreased by 50 per cent or more, or apnoea-hypopnoea index less than 5. The rate of obstructive sleep apnoea resolution (obstructive apnoea-hypopnoea index < 1 event per hour) was also included.

### Statistical methods

Pre- and post-polysomnographic data were compared using paired *t*-tests to examine changes over the course of treatment. Two-sample *t*-tests were used to compare the average change in each polysomnographic parameter over the course of treatment between groups (between-group *p*-value). Because of some missing values, each test was conducted with complete case analysis. All statistical analyses were performed with Stata® 15.1 (a *p*-value of < 0.05 was considered significant in all tests).

## Results and analysis

Ninety-eight patients with endoscopic evidence of laryngomalacia and confirmed obstructive sleep apnoea were initially identified. Sixty-eight patients were excluded because they underwent other surgical treatments (*n* = 32) or did not have evidence of post-treatment polysomnograms (*n* = 36). A flow chart of the process of study identification with inclusion and exclusion criteria is shown in Figure 1.

### Demographics

The average age of obstructive sleep apnoea diagnosis was 13.28 months, with a standard deviation (SD) of ± 14.22 for the surgical group and 13.42 months (SD ± 9.37) for the non-surgical treatment group (*p* = 0.9). There was no statistically significant difference based on ethnicity or sex (*p* = 0.4, *p* = 0.6) (Table 1). Co-morbidities in patients with laryngomalacia, although not uncommon, were not grounds for exclusion from the study, and were relatively similar among both groups. In our cohort, three patients had congenital genetic disorders, specifically two in the supraglottoplasty group (Prader–Willi syndrome and L1CAM syndrome) and one in the non-surgical treatment group (Prader–Willi syndrome). Other co-morbidities included pre-term delivery, hypotonia and gastro-oesophageal reflux. A secondary airway lesion, which was identified in 27.8 per cent of the surgical group at the time of surgery, included laryngeal clefts (16.7 per cent), subglottic stenosis (5.6 per cent) and tracheomalacia (11.1 per cent). Eleven of the 12 (92 per cent) patients treated without surgical intervention were started on single or combined therapy that included nasal steroids (Flonase 50 μg/once daily), montelukast (4 mg daily), omeprazole (1 mg/kg daily), famotidine (0.5 mg/kg twice daily) and/or ranitidine (5 mg/kg daily).

**Table 1.** Demographic characteristics in patients with laryngomalacia and obstructive sleep apnoea (OSA) with secondary airway lesions (SGP)* (*n* = 18) versus non-surgical management (*n* = 12)

Characteristics	SGP (n (%))	Non-surgical management (n (%))	*p*-value
Sex			
Male	12 (66.7)	7 (58)	
Female	6 (33.3)	5 (42)	0.6
Race			
White	10 (55.6)	3 (25)	
African American	4 (22.2)	6 (50)	
Hispanic	2 (11.1)	2 (17)	
Asian	1 (5.6)	1 (8)	
Other	1 (5.6)	0 (0)	0.4
Total	18 (100)	12 (100)	
Co-morbidities			
Craniofacial abnormalities	2 (11.1)	1 (8.3)	0.5
Pre-term delivery	5 (27.8)	2 (16.6)	0.5
Failure to thrive	3 (16.7)	2 (16.6)	0.9
Secondary airway lesions*	5 (27.8)	3 (25)	0.9
Gastro-oesophageal reflux	5 (27.8)	5 (41.6)	0.9
Hypotonia	2 (11.1)	0 (0)	0.5
None	8 (44.4)	2 (16.6)	0.1
Mean age in months of OSA diagnosis (±SD)	13.28 (±14.22)	13.42 (±9.37)	0.9
Days (months) between polysomnographies	494 (16.24)	592 (19.46)	0.9

* SGP: laryngeal cleft = 3, subglottic stenosis = 1, racheomalacia = 1; SD = standard deviation

### Mild obstructive sleep apnoea

In the supraglottoplasty treatment group, four patients had mild obstructive sleep apnoea. Patients with mild obstructive sleep apnoea had an average pre-supraglottoplasty obstructive apnoea-hypopnoea index of 3.98 events per hour and a post-supraglottoplasty obstructive apnoea-hypopnoea index score of 2.4 events per hour for a total average reduction of 1.58 events per hour (*p* = 0.026) (Table 2).

**Table 2.** Pre- and post-treatment PSG measures in the SGP (*n* = 18) and NST (*n* = 12) groups

Average PSG parameter	SGP group pre-treatment	SGP group post-treatment	SGP group *p*-value (CI 95%)	NST group pre-treatment	NST group post-treatment	NST group *p*-value (CI 95%)	Between groups *p*-value (CI 95%)
oAHI (events/hour)	16.31	3.63	0.0039*	7.7	4.4	0.3	0.09
O**_2_** nadir (%)	86.7	87.8	0.4991	86	85	0.7	0.5
Central apnoea (events/hour)	1.38	1.22	0.9	1.04	1.3	0.5	0.6
Weight percentile (%)	40.47	58.52	0.024**	45.9	67.8	0.04**	0.8

*p-value <0.05; PSG = polysomnography; SGP = supraglottoplasty; CI = confidence interval; NST = non-surgical treatment; oAHI = obstructive apnoea-hypopnoea index

Six patients in the non-surgical treatment group had mild obstructive sleep apnoea. This group had a small increase in the number of events from 3.45 events per hour to 5.25 events per hour (*p* = 0.5). Some children who had non-surgical treatment went from mild to moderate or severe obstructive sleep apnoea. The difference between the two treatment groups’ average reduction obstructive apnoea-hypopnoea index scores was not statistically significant (surgery *vs* non-surgical treatment, *p* = 0.3) (Table 2).

**Figure 2.**
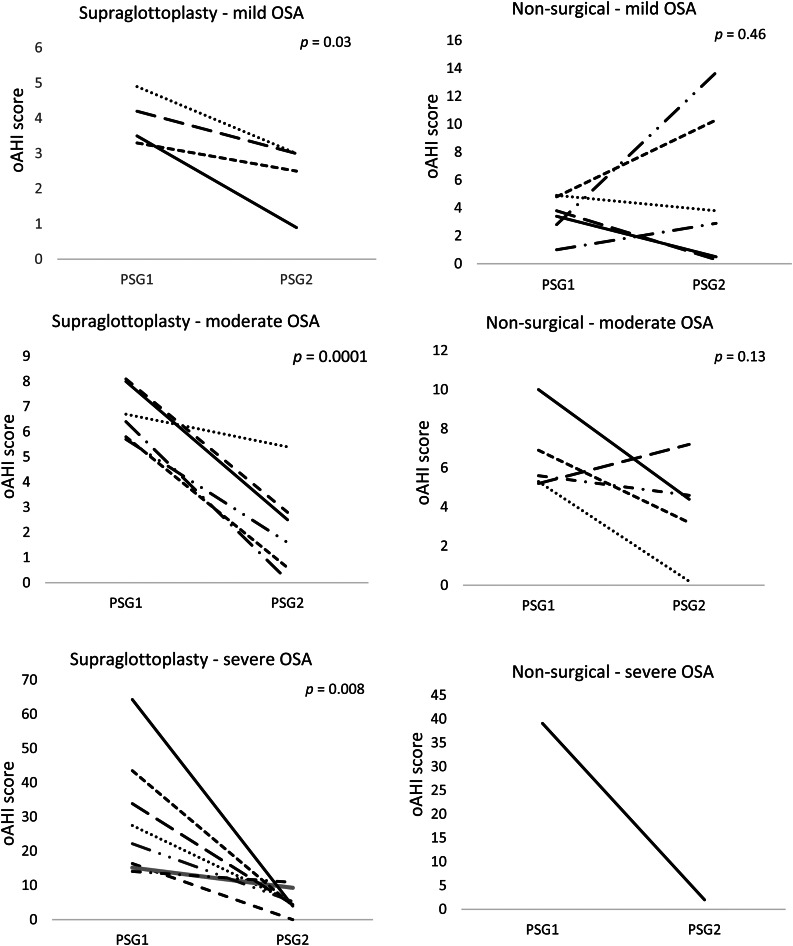
Polysomnography (PSG) versus obstructive apnoea-hypopnoea index (oAHI) change by treatment group based on initial obstructive sleep apnoea (OSA) severity.

All individuals who received a supraglottoplasty had an improvement in the obstructive apnoea-hypopnoea index after surgery but results in the non-surgical treatment group were more variable (Figure 2). Of the four patients in the supraglottoplasty group, one (25 per cent) had resolution of obstructive sleep apnoea (apnoea-hypopnoea index < 1). In the non-surgical treatment group, two of six individuals (33 per cent) had resolution of obstructive sleep apnoea. The O_2_ nadir and weight percentile did not show statistically significant changes in either treatment group. There was a notable reduction in the central apnoea component in the non-surgical treatment group (*p* = 0.03). The significance of this finding is uncertain.

### Moderate obstructive sleep apnoea

Six patients had moderate obstructive sleep apnoea in the supraglottoplasty group. Patients had an average pre-treatment score of 6.8 events per hour and a post-treatment score of 2.2 events per hour, with an average reduction of 4.58 events per hour (*p* < 0.01) (Table 2). All patients treated with supraglottoplasty had improvement in their apnoea and only one child was found to have residual moderate obstructive sleep apnoea after surgery (Figure 2).

Five patients with moderate obstructive sleep apnoea underwent non-surgical treatment. Although there was an average reduction in the obstructive apnoea-hypopnoea index from 6.6 events per hour to 3.9 events per hour, the results were mixed and not significant (*p* = 0.13).

Two of the six (33 per cent) patients in the supraglottoplasty group experienced resolution of obstructive sleep apnoea (apnoea-hypopnoea index < 1) and five of the six had successful surgical outcomes, with an apnoea-hypopnoea index reduction less than 50 per cent. In the non-surgical treatment group for moderate obstructive sleep apnoea, only one of five individuals (20 per cent) had full resolution of obstructive sleep apnoea. The difference between the two treatment groups’ average reduction in obstructive apnoea-hypopnoea index scores was not statistically significant (*p* = 0.2). All other polysomnogram metrics were not statistically significant (Table 3).

**Table 3.** Pre- and post-treatment PSG oAHI in the SGP and NST groups by individual categories of OSA

Average PSG parameter	SGP group pre-treatment	SGP group post-treatment	SGP group *p*-value	NST group pre-treatment	NST group post-treatment	NST group *p*-value	Between groups *p*-value
oAHI (events/hour) mild OSA	3.98	2.4	0.026*	3.45	5.25	0.5	0.3
oAHI (events/hour) moderate OSA	6.8	2.2	<0.001*	6.6	3.9	0.1	0.2
oAHI (events/hour) severe OSA	29.6	5.4	<0.01*	39.1	2	—	—

PSG = polysomnography; SGP = supraglottoplasty; NST = non-surgical treatment; oAHI = obstructive apnoea-hypopnoea index; OSA = obstructive sleep apnoea

### Severe obstructive sleep apnoea

Eight patients with severe obstructive sleep apnoea underwent supraglottoplasty. Patients in this category had an average pre-treatment obstructive apnoea-hypopnoea index score of 29.6 events per hour and an average post-treatment score of 5.4 events per hour, with an average reduction in obstructive apnoea-hypopnoea index score of 24.2 events per hour (*p* < 0.01). All patients had more than 50 per cent obstructive apnoea-hypopnoea index reduction after supraglottoplasty, and one patient had complete obstructive sleep apnoea resolution (Figure 2). The O_2_ nadir and weight percentile increased but were not statistically significant.

There was only one patient with severe obstructive sleep apnoea in the non-surgical treatment group. This patient had a marked improvement in the obstructive apnoea-hypopnoea index from 39.1 events per hour to 2 events per hour.

### All categories of obstructive sleep apnoea combined

The average time between pre-treatment and post-treatment polysomnograms was 494 days (16.24 months) for the supraglottoplasty group and 592 days (19.46 months) for the non-surgical treatment group (Table 1). For all categories of obstructive sleep apnoea together, the supraglottoplasty group had an average obstructive apnoea-hypopnoea index reduction of 12.68 events per hour (polysomnogram 1: 3.3–64.3 events per hour; polysomnogram 2: 0–10.9 events per hour) (*p* = 0.0039), and the non-surgical treatment group had an average reduction of 3.3 events per hour (polysomnogram 1: 1–39 events per hour; polysomnogram 2: 0.2–13.7 events per hour) (*p* = 0.3). Despite the difference above, when compared directly, supraglottoplasty was not superior to conservative treatment (*p* = 0.09) (Table 3).

Patients treated with supraglottoplasty had a 15 per cent increase in their weight percentile (*p* = 0.024), while patients treated without surgery had an average increase of 21.9 per cent (*p* = 0.04) in weight percentile. The difference between the two treatment groups was not statistically significant (*p* = 0.8). The remaining metrics analysed, including O_2_ nadir and central apnoea, were not statistically significant (Table 3).

## Discussion

To our knowledge, this is the first study that compares the treatment outcomes of supraglottoplasty versus conservative therapy in patients with congenital laryngomalacia stratified by obstructive sleep apnoea severity. Our data show that patients treated with supraglottoplasty had a more consistent reduction in obstructive sleep apnoea severity compared to patients treated without surgery (Figure 2). However, there was no statistically significant difference between the treatment groups to suggest that supraglottoplasty is superior to no surgery.

When all severity groups were combined, patients in our cohort had a statistically significant reduction in the obstructive apnoea-hypopnoea index after supraglottoplasty, although residual mild to moderate obstructive sleep apnoea persisted in some children (Figure 2). A possible explanation is that the level and site of obstruction varied among patients in our sample cohort, and was not solely in the larynx (e.g. base of tongue, velopharynx, pharyngeal walls, etc.). Our study group also included patients in both groups with co-morbid conditions such as gastro-oesophageal reflux, hypotonia, and craniofacial anomalies, which can all be associated with obstructive sleep apnoea. Multimodal treatment approaches, including drug-induced sleep endoscopy and drug-induced sleep endoscopy-directed surgery, may be necessary for patients with residual obstructive symptoms.^23^

Using the paediatric polysomnogram severity classification criteria, we attempted to compare the best management modality (supraglottoplasty *vs* conservative treatment) for patients with laryngomalacia and mild, moderate or severe obstructive sleep apnoea.^24^ Our data revealed that supraglottoplasty was beneficial and resulted in a statistically significant reduction in obstructive apnoea-hypopnoea index in patients with mild (*p* = 0.026), moderate (*p* < 0.001) and severe (*p* < 0.001) obstructive sleep apnoea (Figure 2).

When defining surgical success using the traditional definition described by Chang *et al*. (in paediatric sleep apnoea surgery, an obstructive apnoea-hypopnoea index reduction of more than 50 per cent or apnoea-hypopnoea index less than 5), only one patient in the mild supraglottoplasty group was found to meet the criterion. However, when compared to the non-surgical treatment group, all patients treated surgically had a consistent reduction in the obstructive apnoea-hypopnoea index (Figure 2).^25^ In our cohort, the average apnoea-hypopnoea index reduction in these patients was 1.58 events per hour (*p* = 0.026). A recent case-control study evaluated the outcomes of laryngomalacia with observation in 26 children less than 3 years old with mild obstructive sleep apnoea.^19^ The authors of the study reported a significant reduction in the apnoea-hypopnoea index from 2.7 events per hour to 1.3 events per hour (*p* = 0.013), but only 31 per cent had resolution of symptoms. These findings support the conclusion that both observation and surgery can be considered as acceptable therapies for children with laryngomalacia and mild obstructive sleep apnoea.

Patients with moderate and severe obstructive sleep apnoea had a significant and consistent reduction in obstructive apnoea-hypopnoea index after surgery (Figure 2). Eighty-three percent of children with moderate obstructive sleep apnoea and 100 per cent of children with severe obstructive sleep apnoea had more than 50 per cent reduction in the obstructive apnoea-hypopnoea index after supraglottoplasty. Our data showed no statistically significant difference (or superiority) in treatment outcomes between supraglottoplasty and observation/medical management in patients with laryngomalacia and moderate obstructive sleep apnoea (*p* = 0.20).

In a previous study published by Powitzky *et al*., the authors evaluated 20 patients less than 1 year of age with laryngomalacia undergoing supraglottoplasty and found that the obstructive sleep apnoea improved in patients with an initial apnoea-hypopnoea index greater than 5, while infants with an apnoea-hypopnoea index less than 5 had worse polysomnogram values post-operatively.^20^ These results support the use of supraglottoplasty in patients with laryngomalacia and moderate to severe obstructive sleep apnoea. However, their analysis was not stratified by obstructive sleep apnoea severity and lacked a non-surgical comparison group. Our study is the first to compare the effectiveness of supraglottoplasty versus non-surgical treatment in children with congenital laryngomalacia and mild or moderate obstructive sleep apnoea.

As shown in Figure 2, only one patient in our cohort had severe obstructive sleep apnoea and was managed conservatively with anti-reflux medication alone. This patient was initially considered for surgery, but the family opted for conservative therapy because symptoms seemed to be improving over time. A repeat polysomnogram 23 months later showed marked improvement in the obstructive apnoea-hypopnoea index from 39.1 events per hour to 2 events per hour. Chart review of this patient's record did not reveal any additional clinical factors that accounted for the degree of severity initially or the resolution.

- The natural course of obstructive sleep apnoea in children with laryngomalacia is unknown and factors affecting treatment outcomes in children with dual conditions are not thoroughly understood
- This study compared the outcomes of surgical management with supraglottoplasty versus non-surgical management in children with a diagnosis of laryngomalacia and different degrees of obstructive sleep apnoea severity
- In all severity groups combined, patients had a statistically significant reduction in the obstructive apnoea hypopnoea index after supraglottoplasty, but residual mild to moderate obstructive sleep apnoea persisted in some children
- Patients treated with supraglottoplasty had a more consistent and uniform reduction in the obstructive sleep apnoea severity compared to patients treated without surgery
- Patients with laryngomalacia and moderate or severe obstructive sleep apnoea had the greatest benefit from supraglottoplasty, however our analysis did not reveal a statistically significant difference between the treatment groups to suggest that supraglottoplasty is superior to non-surgical management alone
- Supraglottoplasty is an effective treatment modality for patients with moderate and severe obstructive sleep apnoea, in addition children with laryngomalacia and mild obstructive sleep apnoea could also potentially benefit from supraglottoplasty, but to a lesser degree

Our demographic data are similar to other studies on laryngomalacia reporting a Caucasian-predominant population with a male predilection.^18,26,27^ Half (50 per cent) of the non-surgical treatment group was black, while 56 per cent of patients who underwent supraglottoplasty were white (*p* = 0.4). Both groups had similar rates of co-morbidities and secondary airway lesions (Table 1), which made our group more homogeneous. Future studies are needed to identify if race plays a role in the resolution of obstructive sleep apnoea in laryngomalacia, or if there are inequities in the way families are counselled about the different treatment options for laryngomalacia in children.

Although the current study has unique features not previously described in the literature, there are several limitations to consider. First, while the sample size in the supraglottoplasty group is average when taken in the context of similar previous studies, the overall number of patients in each subgroup of obstructive sleep apnoea severity remains small, and conclusions should be interpreted cautiously. Unfortunately, many patients who underwent conservative treatment without surgery had to be excluded from analysis due to absence of a second polysomnogram after a period of conservative therapy. The possibility for selection bias also exists since treatment of choice was not exclusively based on polysomnogram findings, but also on clinical symptomatology. It is possible that children with polysomnogram findings of mild or moderate obstructive sleep apnoea underwent supraglottoplasty based on the severity of symptoms at the time of evaluation. The reverse applies to patients treated conservatively. A prospective, double-blind randomised clinical trial would help eliminate some of these sources of bias but may raise ethical concerns regarding withholding treatment in symptomatic patients.

## Conclusion

This study fills a gap in the literature comparing the efficacy of supraglottoplasty versus non-surgical management in patients with obstructive sleep apnoea and laryngomalacia. Our results suggest that supraglottoplasty is likely an effective treatment modality for patients with moderate and severe obstructive sleep apnoea. Children with laryngomalacia and mild obstructive sleep apnoea potentially could benefit from supraglottoplasty, but to a lesser degree. Although patients treated conservatively also had improvement in their sleep apnoea, the outcomes were more heterogeneous, irregular and not statistically significant across the sleep apnoea severities when compared to supraglottoplasty. Despite this, there was no statistically significant difference between the supraglottoplasty and the non-surgical treatment groups to suggest that surgery is superior. Even though supraglottoplasty is generally considered effective, it is not without risks, and shared decision making may be useful when deciding treatment of choice. Further research with a larger sample population is recommended to validate the findings from this study.
